# Causal relationship between particulate matter 2.5 and infectious diseases: A two-sample Mendelian randomization study

**DOI:** 10.1016/j.heliyon.2023.e23412

**Published:** 2023-12-08

**Authors:** Youjie Zeng, Ke Pang, Si Cao, Guoxin Lin, Juan Tang

**Affiliations:** a Department of Anesthesiology, Third Xiangya Hospital, Central South University, Changsha, Hunan, 410013, China; b Department of Nephrology, Third Xiangya Hospital, Central South University, Critical Kidney Disease Research Center of Central South University, Changsha, 410013, China

**Keywords:** PM_2.5_, Severe acute respiratory syndrome coronavirus, Mendelian randomization, Causal relationship, Risk factors

## Abstract

**Background:**

Previous observational studies suggested a correlation between particulate matter 2.5 (PM_2.5_) and infectious diseases, but causality remained uncertain. This study utilized Mendelian randomization (MR) analysis to investigate causal relationships between PM_2.5_ concentrations and various infectious diseases (COVID-19 infection, hospitalized COVID-19, very severe COVID-19, urinary tract infection, bacterial pneumonia, and intestinal infection).

**Methods:**

Inverse variance weighted (IVW) was the primary method for evaluating causal associations. For significant causal estimates, multiple sensitivity tests were further performed: (i) three additional MR methods (MR-Egger, weighted median, and maximum likelihood method) for supplementing IVW; (ii) Cochrane's Q test for assessing heterogeneity; (iii) MR-Egger intercept test and MR-PRESSO global test for evaluating horizontal pleiotropy; (iv) leave-one-out sensitivity test for determining the stability.

**Results:**

PM_2.5_ concentration significantly increased the risk of hospitalized COVID-19 (OR = 1.91, 95 % CI: 1.06–3.45, *P* = 0.032) and very severe COVID-19 (OR = 3.29, 95 % CI: 1.48–7.35, *P* = 3.62E-03). However, no causal effect was identified for PM_2.5_ concentration on other infectious diseases (*P* > 0.05). Furthermore, various sensitivity tests demonstrated the reliability of significant causal relationships.

**Conclusions:**

Overall, lifetime elevated PM_2.5_ concentration increases the risk of hospitalized COVID-19 and very severe COVID-19. Therefore, controlling air pollution may help mitigate COVID-19 progression.

## Teaser

**Summary:** This study employed Mendelian randomization analysis to investigate the impact of PM2.5 on infectious diseases. Elevated PM2.5 levels were found to significantly increase the risk of hospitalization and severe COVID-19, highlighting the importance of air pollution control in mitigating COVID-19.

## 1 Introduction

Coronavirus disease 2019 (COVID-19) has emerged as a global pandemic caused by the severe acute respiratory syndrome coronavirus-2 (SARS-CoV-2) [1]. Controlling the spread of this virus presents a significant challenge, given that approximately 80 % of individuals infected with SARS-CoV-2 remain asymptomatic or exhibit only mild symptoms [2,3]. Conversely, a subset of patients experiences severe manifestations of the infection, including coughing, dyspnea, respiratory failure, and subsequent hospitalization in intensive care units, or even death [4]. Certain demographic and medical factors, such as advanced age, obesity, and underlying conditions like diabetes, cardiovascular disease, chronic kidney disease, or chronic respiratory disease, render individuals more susceptible to developing severe COVID-19 [[5], [6], [7]]. However, these factors alone may not sufficiently explain the observed seasonal variation in COVID-19 prevalence [8]. Consequently, our investigation has been directed toward exploring environmentally linked risk factors, with a specific focus on air pollution.

Particulate matter 2.5 (PM_2.5_) constitutes a significant air pollutant, functioning as a micro-reservoir for various air pollutants, including organic pollutants and heavy metal ions, thereby posing a serious threat to human health [9,10]. Substantial evidence supports the notion that exposure to PM_2.5_ pollution may enhance susceptibility to, and the severity of, viral or bacterial infectious diseases [11,12]. Previous studies have suggested a link between prolonged exposure to PM_2.5_ and COVID-19 morbidity and mortality [13,14]; nevertheless, this correlation remains a topic of controversy [15]. A nationwide cross-sectional study conducted in the United States found no significant link between COVID-19 case fatality and prolonged exposure to PM_2.5_ [16]. Furthermore, investigations have established a link between elevated PM_2.5_ levels and bacterial infections, such as Streptococcus pneumoniae pneumonia, urinary tract infections, and gastrointestinal disorders [[17], [18], [19], [20], [21]]. To date, the causative relationship between PM_2.5_ exposure and infectious diseases remains unclear, necessitating more robust evidence for support.

Mendelian randomization (MR) is increasingly recognized as a reliable approach for establishing causal links between exposures and disease outcomes, utilizing genetic variants as instrumental variables (IVs) [22]. The introduction of genome-wide association studies (GWAS) has greatly augmented the repertoire of identified genetic variants associated with complex exposures, thereby catalyzing the widespread utilization of MR to an unprecedented extent [23]. Furthermore, a strength of MR studies lies in their ability to assess the causal impact of varying lifelong exposure levels on outcomes [24]. In this study, we utilized a two-sample MR strategy to assess the causal associations between PM_2.5_ exposure and various infectious diseases (COVID-19 infection, hospitalized COVID-19, very severe COVID-19, urinary tract infection, bacterial pneumonia, and intestinal infection).

## 2 Materials and methods

### 2.1 Data sources for GWAS summary statistics

Details of all GWAS summary statistics available for this MR study are shown in Table 1. According to previous studies [25,26], GWAS summary statistics for PM_2.5_ were obtained from the IEU OpenGWAS project database [27]. The dataset included 423,796 European individuals with PM_2.5_ concentration of 9.99 (±1.06) μg/m^3^.

**Table 1:** Information of GWAS summary statistics for MR analysis.

Characteristics	Sample size	Data sources	Ancestry	Data download
PM_2.5_	423,796	MRC-IEU	European	https://gwas.mrcieu.ac.uk/datasets/ukb-b-10817/
COVID-19 infection	Number of cases: 122,616 Number of controls: 2,475,240	COVID-19 host genetics initiative	European	https://www.covid19hg.org/results/r7/
Hospitalized COVID-19	Number of cases: 32,519 Number of controls: 2,062,805	COVID-19 host genetics initiative	European	https://www.covid19hg.org/results/r7/
Very severe COVID-19	Number of cases: 13,769 Number of controls: 1,072,442	COVID-19 host genetics initiative	European	https://www.covid19hg.org/results/r7/
Urinary tract infection	Number of cases: 12,491 Number of controls: 379,936	Lee Lab	European	https://pheweb.org/UKB-SAIGE/pheno/591
Bacterial pneumonia	Number of cases: 6,710 Number of controls: 398,538	Lee Lab	European	https://pheweb.org/UKB-SAIGE/pheno/480.1
Intestinal infection	Number of cases: 8,991 Number of controls: 399,970	Lee Lab	European	https://pheweb.org/UKB-SAIGE/pheno/008

**Abbreviations:** PM2.5: particulate matter 2.5.

GWAS summary statistics for COVID-19-related traits were obtained from a large-scale GWAS meta-analysis (release 7) conducted by the COVID-19 Host Genetics Initiative [28]. Three relevant phenotypes were included: (i) COVID-19 infection (122,616 cases and 2,475,240 controls), (ii) hospitalized COVID-19 (32,519 cases and 2,062,805 controls), and (iii) very severe COVID-19, which refers to individuals experiencing outcomes such as death, mechanical ventilation, non-invasive ventilation, high-flow oxygen requirement, or the utilization of extracorporeal membrane oxygenation (13,769 cases and 1,072,442 controls).

In addition, GWAS summary statistics for urinary tract infection (12,491 cases and 379,936 controls), bacterial pneumonia (6,710 cases and 398,538 controls), and intestinal infection (8,991 cases and 399,970 controls) defined according to the International Classification of Diseases were obtained from Lee Lab (https://www.leelabsg.org/resources).

### 2.2 Selection of IVs

IVs selection was performed according to three core assumptions of the MR study [29]: (i) IVs are highly correlated with exposure; (ii) IVs are not associated with confounders that influence exposure and outcome; (iii) IVs can only affect the outcome through exposure and are not directly associated with outcome.

To comply with core assumption 1, SNPs that fulfilled the genome-wide significance threshold (*P* < 5e-8) were screened, followed by the exclusion of SNPs in linkage disequilibrium (threshold of r^2^ < 0.001 within 10,000 kb).

To comply with core assumption 2, all traits associated with the remaining IVs were queried through the PhenoScanner V2 database [30]. Any SNPs associated with confounders would be removed from the IVs.

To comply with core assumption 3, the correlation between IVs with exposure should be stronger than the correlation with outcomes before performing MR analysis. In addition, various sensitivity tests were performed to assess horizontal pleiotropy.

Finally, *F*-statistics were calculated for each IV, and only IVs with *F*-statistics greater than 10 were retained, thus mitigating bias caused by weak IVs [31].

### 2.3 Statistical methods

**Fig. 1:**
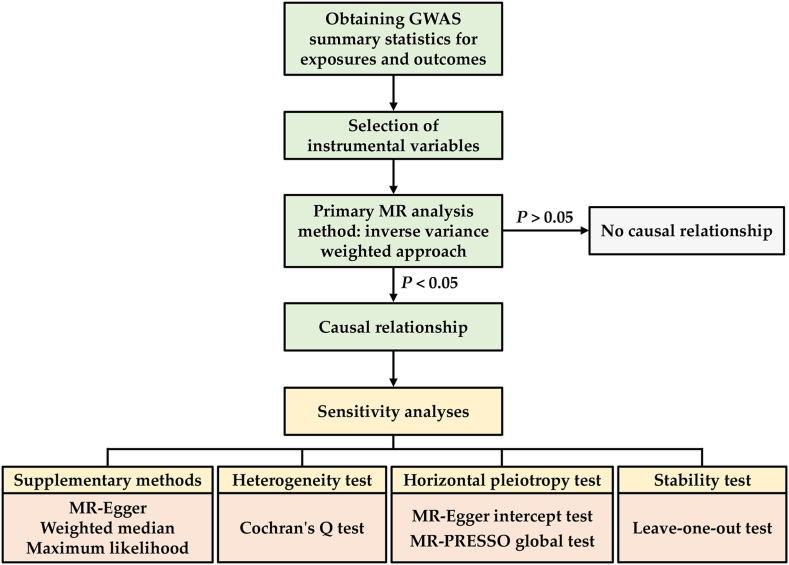
Overall analysis flow of this study.

The overall analysis flow of this study is shown in Fig. 1. The primary method for evaluating causal associations was the inverse variance weighted (IVW) approach. This method initially evaluated the causal impact of the exposure on the outcome using the Wald ratio approach for each individual IV. Subsequently, a meta-analysis was conducted using fixed or random effects models [32]. Given that the outcomes were binary variables, the causal estimates were reported in terms of odds ratios (OR) along with corresponding 95 % confidence intervals (CI). Statistical significance was determined at a threshold of *P* < 0.05.

Furthermore, additional sensitivity analyses were conducted to validate the significant estimates obtained from the primary MR analysis. Firstly, three supplementary methods (MR-Egger, weighted median, and maximum likelihood method) were employed to complement the IVW approach. MR-Egger yields reasonable estimates even in the presence of horizontal pleiotropy [33]. Weighted medians can provide reliable estimates when only half of the IVs are valid [34]. The maximum likelihood method maximizes the likelihood function to estimate the parameters of the probability distribution with minimal standard errors [35]. Subsequently, heterogeneity was assessed using Cochran's Q test, which examines the variance in causal effects estimated by different IVs. Additionally, the MR-Egger intercept test and the MR-PRESSO global test were employed to evaluate horizontal pleiotropy, offering further evidence regarding the fulfillment of the third MR core assumption. Lastly, the leave-one-out test involved replicating the MR analysis using the remaining IVs after excluding each IV, thereby investigating whether any influential SNPs significantly affected the overall results.

The statistical analyses were conducted in R software (version 4.3.1) utilizing primarily the “TwoSampleMR” R package and the “MR-PRESSO” R package.

## 3 Results

### 3.1 Identification of IVs

Eight SNPs associated with PM_2.5_ were identified as IVs under the genome-wide significance threshold and after excluding SNPs in linkage disequilibrium (Supplementary Table S1). The PhenoScanner V2 database indicated that these SNPs were not significantly related to potential confounders (Supplementary Table S2). In addition, the *F*-statistics of all IVs were >10 (30.04–69.92). Details of the IVs used for MR analysis are shown in Supplementary Table S3.

### 3.2 Causal effect of PM_2.5_ on infectious diseases

**Fig. 2:**
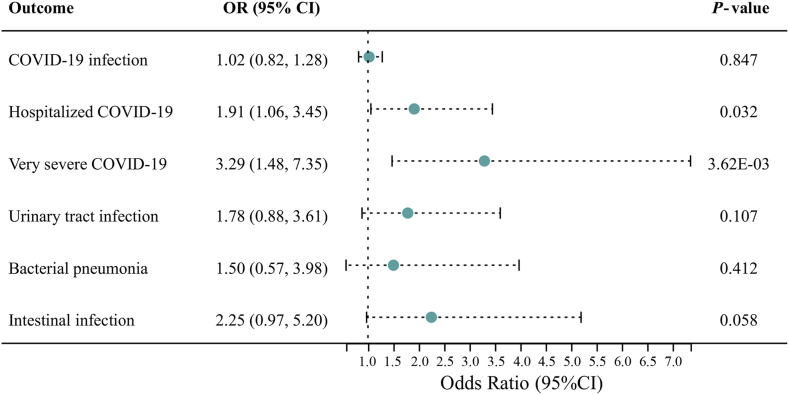
Identification of the causal effects of PM_2.5_ on infectious diseases by IVW MR method. Abbreviations: OR: odds ratio; CI: confidence interval.

As shown in Fig. 2, the primary MR results by IVW showed a significant positive causal effect of PM_2.5_ on hospitalized COVID (OR = 1.91, 95 % CI: 1.06–3.45, *P* = 0.032) and very severe COVID-19 (OR = 3.29, 95 % CI: 1.48–7.35, *P* = 3.62E-03). However, the IVW method indicated that PM_2.5_ would not causally affect COVID-19 infection (OR = 1.02, 95 % CI: 0.82–1.28, *P* = 0.847), urinary tract infection (OR = 1.78, 95 % CI: 0.88–3.61, *P* = 0.107), bacterial pneumonia (OR = 1.50, 95 % CI: 0.57–3.98, *P* = 0.412), and intestinal infection (OR = 2.25, 95 % CI: 0.97–5.20, *P* = 0.058).

### 3.3 Sensitivity tests indicated the reliability of MR results

**Fig. 3:**
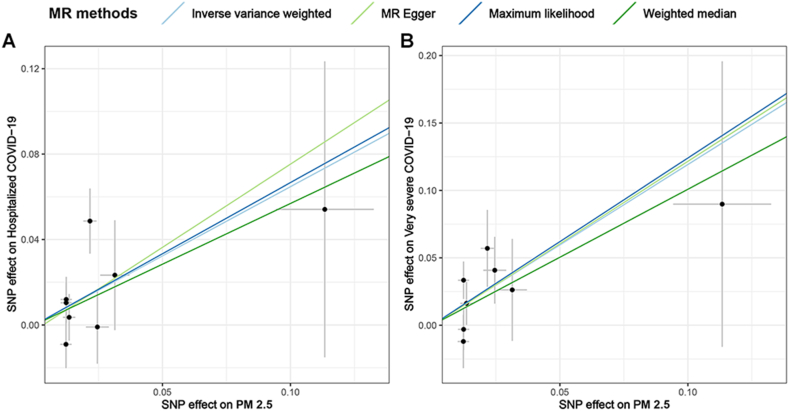
Scatter plot of MR results by diverse methods. (A) Scatter plot of SNP effects on PM_2.5_ versus hospitalized COVID-19. (B) Scatter plot of SNP effects on PM_2.5_ versus very severe COVID-19.

**Fig. 4:**
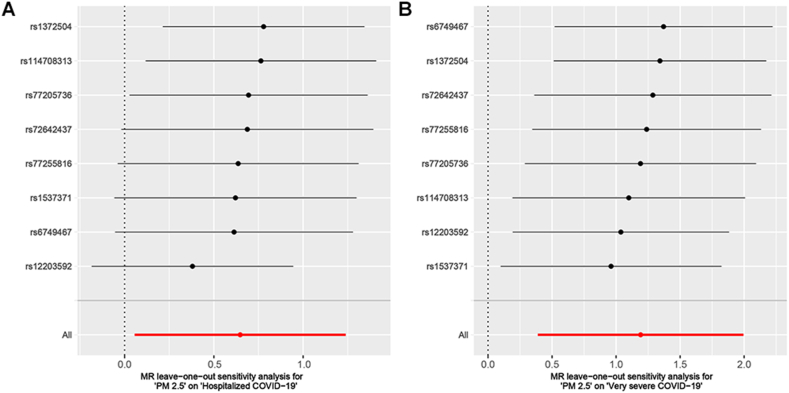
Results of the leave-one-out sensitivity analysis. (A) MR leave−one−out sensitivity analysis for ‘PM_2.5_' on ‘hospitalized COVID-19'. (B) MR leave−one−out sensitivity analysis for ‘PM_2.5_' on ‘very severe COVID-19'.

The causal effects of PM_2.5_ on hospitalized COVID and very severe COVID-19 were further demonstrated using various sensitivity tests. Firstly, the results of MR-Egger, weighted median, and maximum likelihood method are parallel to IVW (OR > 1) (Supplementary Table S4 and Fig. 3). Secondly, Cochran's Q test indicated no heterogeneity among the IVs used for MR analysis: [(i) PM_2.5_ and hospitalized COVID-19: Q_pval_IVW_ = 0.256, Q_pval_MR–Egger_ = 0.180; (ii) PM_2.5_ and very severe COVID-19: Q_pval_IVW_ = 0.464, Q_pval_MR–Egger_ = 0.353] (Supplementary Table S5). Thirdly, the MR-Egger intercept test and MR-PRESSO global test showed that MR analysis was not affected by significant horizontal pleiotropy [MR-Egger intercept test: (i) PM_2.5_ and hospitalized COVID-19: intercept = −2.61E-03, *P* = 0.835; (ii) PM_2.5_ and very severe COVID-19: intercept = −5.83E-04, *P* = 0.972. MR-PRESSO global test: (i) PM_2.5_ and hospitalized COVID-19: RSS obs = 11.785, *P* = 0.281; (ii) PM_2.5_ and very severe COVID-19: RSS obs = 8.373, *P* = 0.524] (Supplementary Table S6). Finally, the leave-one-out test revealed the stability of the MR results, as removing any IV did not significantly alter the MR results (Fig. 4).

## 4 Discussion

In the present study, we conducted a two-sample MR analysis using extensive GWAS datasets to investigate the causal relationship between PM_2.5_ exposure and infectious diseases, including COVID-19, urinary tract infection, bacterial pneumonia, and intestinal infection. Our findings revealed a significant association between elevated PM_2.5_ levels and an increased risk of hospitalized COVID-19 and very severe COVID-19. However, no causal effect was identified for PM_2.5_ concentration on COVID-19 infection, urinary tract infection, bacterial pneumonia, and intestinal infection. It should be noted that this study represents the first large-scale application of MR analysis to assess the causal relationship between PM_2.5_ exposure and infectious diseases. This significantly enriches and enhances the existing evidence of related investigations.

In previous studies, inconsistencies in the association between PM_2.5_ and COVID-19 have been noted. Specifically, a study conducted in Ohio discovered an increased risk of COVID-19 hospitalization among individuals exposed to PM_2.5_ (OR: 1.18, 95 % CI: 1.11–1.26) [13]. Likewise, a Dutch study utilizing ecological regression analysis revealed a positive relationship between air pollution, particularly PM_2.5_ concentrations, and the incidence of COVID-19 infections, hospitalizations, and fatalities [36]. Furthermore, a study covering 3,089 counties in the United States revealed a positive association between increased PM_2.5_ exposure and elevated COVID-19 mortality, even after adjusting for various area-level confounding factors [14]. Contrastingly, another national cross-sectional study in the United States found no significant association between PM_2.5_ and COVID-19 morbidity and mortality [16]. These conflicting findings could stem from variations in patient sample sources, confounding factors, and diverse study designs. Conventional observational epidemiological studies are susceptible to confounding factors, which can compromise the reliability of etiological interpretations. To overcome these limitations, this study utilized a two-sample MR approach to examine the causal relationship. The findings remained consistent with traditional observational studies, indicating that elevated PM_2.5_ levels significantly increased the risk of COVID-19 hospitalization (OR = 1.91, 95 % CI: 1.06–3.45, *P* = 0.032) and critical illness (OR = 3.29, 95 % CI: 1.48–7.35, *P* = 3.62E-03). Consequently, efforts to reduce air pollution and lower PM_2.5_ concentrations could be effective in reducing the risk of COVID-19.

Presently, there exists a range of potential biological mechanisms by which PM_2.5_ exacerbates the severity of COVID-19. Within the lungs, PM_2.5_ alters the immune response of the lung cells, resulting in the induction of the release of pro-inflammatory cytokines from activated alveolar macrophages and promoting the production of free radicals due to the presence of heavy metals and polycyclic aromatic hydrocarbons on its surface [37]. Consequently, the excessive production of free radicals diminishes cellular antioxidant capacity, leading to lipid peroxidation and cellular damage, exacerbating lung injuries associated with COVID-19 [38]. Moreover, due to its small size, PM_2.5_ can permeate various organs via the bloodstream, resulting in detrimental health effects [39,40]. Research has shown that PM_2.5_ can impede the expression and activity of innate antioxidant enzymes within the body [41], trigger a heightened oxidative stress response, and impose detrimental effects on various organ systems [42], exacerbating the systemic dysfunction caused by COVID-19.

Previous investigations have reported a correlation between PM_2.5_ exposure and bacterial infections. PM_2.5_, a form of particulate matter, has been linked to alterations in the composition of the pharyngeal microbiota in humans [43]. Studies have shown that elevated concentrations of both PM_2.5_ and PM_10_ host a diverse range of bacterial and archaeal species, including some that are pathogenic or opportunistic pathogens [43]. Exposure to PM_2.5_ has also been associated with an increased occurrence and fatality rate of respiratory infections, including bacterial infections [44]. Chen et al. observed that PM_2.5_ exposure impairs macrophage functions, potentially compromising innate immune defenses against bacterial infections [18]. Nevertheless, a recent study by Karimi et al. found no connection between PM_2.5_ levels and community-associated staphylococcal infections [45]. In our MR analysis, although we observed a positive correlation trend between PM_2.5_ and urinary tract infections, bacterial pneumonia, and intestinal infections, no statistically significant result was identified. This indicates the necessity for larger-scale, specific GWAS datasets to establish conclusive evidence.

This MR study has several limitations that require clarification. Firstly, all the GWAS data included in this study are from individuals of European ancestry, so it is unclear whether the conclusions are applicable to other populations. Secondly, since the present MR study was conducted based on summary-level GWAS statistics, stratified analyses (based on age or gender) cannot be performed. Thirdly, due to the possibility of population relocation, which could lead to changes in PM 2.5 levels, this factor cannot be taken into account in the current study. Finally, despite comprehensive sensitivity testing, it cannot be guaranteed that potential horizontal pleiotropy interference is entirely eliminated.

## 5 Conclusion

Overall, the present MR study indicates that lifetime elevated PM_2.5_ concentration may increase the risk of hospitalized COVID-19 and very severe COVID-19. Therefore, efforts to control air pollution may help mitigate COVID-19 progression.

## Funding

This research was funded by the National Natural Science Foundation for Distinguished Young Scholars of China (No. 81900634 to Dr. Tang), the Natural Sciences Foundation of Hunan Province for Distinguished Young Scholars (No. 2021JJ40947 to Dr. Tang), the Natural Science Foundation of Changsha City (No. kq2208356 to Dr. Lin).

## Patient consent statement

All data analyzed in this study were obtained from publicly available databases in which ethical approval was obtained for each cohort, and informed consent was obtained from all participants prior to participation. Therefore, no additional ethical approval or patient consent was required for this MR study.

## Data availability statement

The sources of the GWAS summary statistics used for this MR study are shown in Table 1.

## CRediT authorship contribution statement

**Youjie Zeng:** Writing - review & editing, Writing - original draft, Visualization, Validation, Resources, Project administration, Methodology, Investigation, Formal analysis, Data curation, Conceptualization. **Ke Pang:** Writing - review & editing, Validation, Methodology, Investigation, Formal analysis, Data curation. **Si Cao:** Writing - review & editing, Validation, Methodology, Investigation, Data curation. **Guoxin Lin:** Validation, Supervision, Resources, Project administration, Methodology, Investigation. **Juan Tang:** Writing - review & editing, Supervision, Resources, Project administration, Methodology, Investigation, Funding acquisition.

## Declaration of competing interest

The authors declare that they have no known competing financial interests or personal relationships that could have appeared to influence the work reported in this paper.
